# Impaired Skeletal Muscle Development and Regeneration in Transglutaminase 2 Knockout Mice

**DOI:** 10.3390/cells10113089

**Published:** 2021-11-09

**Authors:** Zsófia Budai, Nour Al-Zaeed, Péter Szentesi, Hajnalka Halász, László Csernoch, Zsuzsa Szondy, Zsolt Sarang

**Affiliations:** 1 Doctoral School of Molecular Cell and Immune Biology, Faculty of Medicine, University of Debrecen, 4032 Debrecen, Hungary; budai.zsofia@med.unideb.hu (Z.B.); nrzaied@med.unideb.hu (N.A.-Z.); halasz.hajnalka@med.unideb.hu (H.H.); 2 Department of Physiology, Faculty of Medicine, University of Debrecen, 4032 Debrecen, Hungary; szentesi.peter@med.unideb.hu (P.S.); csl@edu.unideb.hu (L.C.); 3 Department of Biochemistry and Molecular Biology, Faculty of Medicine, University of Debrecen, 4032 Debrecen, Hungary; sarang@med.unideb.hu; 4 Division of Dental Biochemistry, Department of Basic Medical Sciences, Faculty of Dentistry, University of Debrecen, 4032 Debrecen, Hungary

**Keywords:** transglutaminase 2, muscle repair, macrophage, myoblast fusion, muscle development

## Abstract

Skeletal muscle regeneration is triggered by local inflammation and is accompanied by phagocytosis of dead cells at the injury site. Efferocytosis regulates the inflammatory program in macrophages by initiating the conversion of their inflammatory phenotype into the healing one. While pro-inflammatory cytokines induce satellite cell proliferation and differentiation into myoblasts, growth factors, such as GDF3, released by healing macrophages drive myoblast fusion and myotube growth. Therefore, improper efferocytosis may lead to impaired muscle regeneration. Transglutaminase 2 (TG2) is a versatile enzyme participating in efferocytosis. Here, we show that TG2 ablation did not alter the skeletal muscle weights or sizes but led to the generation of small size myofibers and to decreased grip force in TG2 null mice. Following cardiotoxin-induced injury, the size of regenerating fibers was smaller, and the myoblast fusion was delayed in the tibialis anterior muscle of TG2 null mice. Loss of TG2 did not affect the efferocytic capacity of muscle macrophages but delayed their conversion to Ly6C^−^CD206^+^, GDF3 expressing cells. Finally, TG2 promoted myoblast fusion in differentiating C2C12 myoblasts. These results indicate that TG2 expressed by both macrophages and myoblasts contributes to proper myoblast fusion, and its ablation leads to impaired muscle development and regeneration in mice.

## 1. Introduction

Extensive mechanical stress frequently causes micro-traumas in skeletal muscle, followed by a regeneration period. This regeneration is composed of three major phases: first, inflammation characterized by leukocyte infiltration to the damage site; second, new tissue formation where quiescent myogenic stem cells, also called satellite cells, become activated, enter myogenesis, proliferate, and differentiate into myoblasts; and third, tissue remodeling phase accompanied by fusion of myoblasts followed by growth of muscle fibers and revascularization [1]. During the inflammation phase, a transient wave of neutrophils’ entry is followed by that of M1 polarized inflammatory macrophages (Mϕ). These Mϕs produce pro-inflammatory mediators, such as interleukin 1 (IL-1), tumor necrosis factor alpha (TNF-α), and nitric oxide, which activate quiescent satellite cells to enter the cell cycle and to start proliferation and differentiation into myoblasts [2,3]. Mϕs clear necrotic myofibers and dying neutrophils, and the efferocytosis process reprograms them to M2-like pro-resolution and healing Mϕs which promote the resolution of inflammation and tissue repair [4]. M2-like Mϕs secrete anti-inflammatory cytokines and lipids, such as IL-10 and resolvins, to suppress inflammation [5,6]. These cells are also the source of growth factors, e.g., the transforming growth factor-β (TGF-β) family member growth differentiation factor 3 (GDF3) that specifically stimulates myogenic cell fusion to build new myofibers [7] and TGF-β that negatively regulates the process [8].

Tissue transglutaminase 2 (TG2) is a multifunctional enzyme with transamidase, protein disulfide isomerase, and guanine and adenine nucleotide-binding and -hydrolyzing activities. In its open conformation, by using its transamidase activity, TG2 establishes covalent crosslinks between proteins or proteins and various biological amines, while in the closed conformation, it functions as a G protein in various intracellular signaling pathways [9]. Extracellularly, it can be found in either in the closed or in a largely unknown conformation bound to cell surface proteins such as Gpr56 [10] or integrin beta 1, 3, and 5 (ITGβ1, 3, and 5) [11,12] interacting also with their ligands, such as fibronectin [11] and MFG-E8 [13], promoting their signaling. Acting so, TG2 has been reported to be involved in a broad variety of biological processes, such as apoptosis [14], fibrosis [15], dead cell phagocytosis [13,16], wound healing [17], and inflammation [18].

TG2 expression is normally undetectable in the postnatal skeletal muscle but was found to be highly expressed by myoblasts during the early embryonic muscle development [19,20], where it was implicated to contribute to the myotube growth and myofibril assembly [21,22]. Its skeletal muscle expression is also increased in idiopathic inflammatory myopathies and during wound healing [17,23], indicating that the biological program of inflammation and tissue repair might involve TG2. Based on its versatile biological roles, we decided to test the involvement of TG2 in muscle development and regeneration using a full-body TG2 knockout mouse strain.

## 2. Materials and Methods

### 2.1. Reagents

All reagents were obtained from Sigma-Aldrich (Budapest, Hungary) except when indicated otherwise.

### 2.2. Experimental Animals

Experiments were carried out using 2–5-month-old full-body knockout C57BL/6 TG2^+/+^ and TG2^−/−^ male mice [24] bred in the heterozygous form under specific pathogen-free conditions in the central animal facility of the University of Debrecen. All animal experiments were approved by the Animal Care and Use Committee of the University of Debrecen (DEMÁB) with permission number 7/2016/DEMÁB.

### 2.3. Cardiotoxin-Induced Muscle Injury Model

Muscle injury was induced as described previously [25]. Briefly, pentobarbital (80 mg/kg mouse) was injected intraperitoneally to anesthetize the mice. Muscle damage was induced in the tibialis anterior (TA) muscle by injecting 50 µL of 12 µM cardiotoxin (Latoxan) in phosphate-buffered saline (PBS). Mice were sacrificed at various time points post-injury, and muscles were collected and processed for immunohistochemical staining, or for cell or mRNA isolation.

### 2.4. Histological Examination of Various Muscles

Histological examination was carried out as described previously [25]. In brief, TA muscles were collected at various time points and preserved for one day in formaldehyde (4% in PBS). Before embedding in paraffin, the tissue was dehydrated as per standard protocol. After the paraffin solidified, the blocks were cut into 6µm thick sections using a microtome. Hematoxylin and eosin (H&E) staining was used after deparaffinization to determine overall morphology and the presence of necrotic fibers following damage. An AMG EVOS fl microscope was used to photograph the sections (Thermo Fisher Scientific, Waltham, MA, USA).

### 2.5. Immunofluorescent Staining

Immunofluorescent staining was carried out as described earlier [25]. Briefly, muscles from control non-injected or CTX-injected mice were removed on days 2, 3, 4, 8, and 16 for histological assessment, and 7 µm thick cryosections were cut at −20 °C using a 2800 Frigocut microtome (Leica, St. Jouarre, France). For the cross-sectional area (CSA) and collagen-stained area calculation, the frozen muscle sections were stained with Dylight 488 conjugated anti-laminin B (cat#: PA5-22901, Invitrogen, Carlsbad, CA, USA) (1:100) and anti-collagen 1 antibody (1:100) followed by Alexa Fluor 488 conjugated goat anti-rabbit IgG secondary antibody. For myosin heavy chain (MYHC) 4 staining methanol-fixed C2C12 cells were incubated with Alexa fluor488 conjugated anti-MYHC4 antibody in 1:100 dilution (cat# 53-6503-82, Thermo Fisher Scientific, Waltham, MA, USA) followed by washing with 4 µg/mL 4′,6-diamidino-2-phenylindole (DAPI) (Invitrogen, Carlsbad, CA, USA) and mounting with glass coverslips. Pictures were taken on a FLoid Cell Imaging Station fluorescent microscope (Thermo Fisher Scientific, Waltham, MA, USA). Images were analyzed using ImageJ version 1.52p software (National Institutes of Health, Bethesda, MD, USA) with a muscle morphometry plugin. Regenerating muscle regions were defined as areas with fibers containing centrally positioned nuclei. CSAs were measured in µm^2^, whereas collagen deposition was expressed as a percentage of the overall regenerating area.

### 2.6. In Vivo Assessment of Muscle Force

The force of the forepaw was measured as described earlier [26]. Briefly, the animals were gently pushed away horizontally from the instrument once they had consistently grabbed the bar of the grip test meter. The maximum force before the animal released the bar was digitized at 2 kHz and saved. To get a single data point for each mouse, the test was repeated 10–15 times. The grip test was measured on the day the animals were sacrificed for all animal groups.

### 2.7. Ex Vivo Assessment of Muscle Force

Muscle contraction was measured as described previously [26]. In brief, the fast-twitch extensor digitorum longus (EDL) and the slow-twitch muscle soleus (SOL) were manually removed and placed horizontally in an experimental chamber continuously superfused (10 mL/min) with Krebs’ solution (containing in mM: NaCl 135, KCl 5, CaCl_2_ 2.5, MgSO_4_ 1, Hepes 10, glucose 10, NaHCO_3_ 10; pH 7.2; room temperature) equilibrated with 95% O_2_ plus 5% CO_2_. The muscle was linked to a rod on one end and to a capacitive mechano-electric force transducer on the other. To induce single twitches, two platinum electrodes were inserted underneath the muscle, which delivered short, supramaximal pulses of 2 ms in duration. The force responses were digitized at 2 kHz using Digidata 1200 A/D card and stored with Axotape software (Axon Instruments, Foster City, CA, USA). Muscles were then stretched by altering the location of the transducer to a length that provided the maximum force response and allowed to equilibrate for 5 min. To induce single twitches, single pulses at 0.5 Hz were employed. Under these settings, at least 10 twitches were recorded from each muscle. Individual force transients within such a train varied in amplitude by less than 3%; hence, the mean of all transients’ amplitudes was utilized to characterize the muscles. Single pulses of 200 Hz frequency for 200 ms (EDL) or 100 Hz for 500 ms (soleus) were used to trigger tetanus. The time between the onset of the transient and the relaxation to 10% of the maximal force was used to calculate the duration of individual twitches and tetani.

### 2.8. Isolation of Muscle-Derived CD45^+^ Leukocytes

Muscle-derived CD45^+^ cell isolation was carried out as described earlier [25]. Briefly, on days 2, 3, 4, and 6 post-injury, TA muscles were removed and dissociated in RPMI with 0.2 percent collagenase II (Thermo Fisher Scientific, Waltham, MA, USA) at 37 °C for 1 h before being filtered through a 100 µm and then a 40 µm filter.

CD45^+^ cells were separated using magnetic sorting (Miltenyi Biotec, Gladbach, Germany).

### 2.9. Gene Expression Analysis

Gene expressions were determined as described earlier [25]. Briefly, total RNA was extracted from TA muscle, C2C12, or muscle-derived CD45^+^ cells using TRIzol (Invitrogen, Carlsbad, CA, USA) reagent according to the manufacturer’s instructions. RNA was reverse transcribed into cDNA using High-Capacity cDNA Reverse Transcription Kit (Life Technologies, Budapest, Hungary) according to the manufacturer’s instructions. Quantitative RT-PCR was carried out in triplicate using pre-designed FAM-labeled MGB assays (Life Technologies, Budapest, Hungary) on a Roche LightCycler LC 480 real-time PCR instrument. Relative mRNA levels were calculated using the comparative C_T_ method and were normalized to beta-actin (β-actin) or Casein Kinase 2 Alpha 2 (Csnk2α2) mRNA. The primers used were the following: Actb Mm02619580_g1, Csnk2a2 Mm00441242_m1, Itgb1 Mm01253230_m1, Itgb3 Mm00443980_m1, Tgfb1 Mm01178820_m1, MyoD1 Mm00440387_m1, Myhc1 Mm01332489_m1, Myog Mm00446194_m1, Tnf Mm00443258_m1, Gdf3 Mm00433563_m1, IL1B Mm00434228_m1, IL10 Mm01288386_m1, IL6 Mm00446190_m1, Arg1 Mm00475988_m1, MFG-E8 Mm00500549_m1, MCP-1 Mm00441242_m1, GPR56 Mm00817704_m1, TG2 Mm00436979_m1, and Pax7 Mm00834082_m1.

### 2.10. Quantification of Intramuscular Immune and Satellite Cells by Flow Cytometry

For intramuscular satellite cell detection, TA were collected at day 2, 3, 4, and 6 post-injury and dissociated in RPMI-1640 medium containing 0.2% collagenase II (Gibco) at 37 °C for 1 h and filtered through a 100 µm. The cell suspensions were stained in two steps for SC detection with the following antibodies: biotin anti-mouse CD45 (103104, BioLegend, San Diego, CA, USA), biotin anti-mouse CD31 (102404, BioLegend, San Diego, CA, USA), biotin anti-mouse Ly-6A/E (Sca1) (122504, BioLegend, San Diego, CA, USA), biotin anti-mouse TER-119/Erythroid cells (79748, BioLegend, San Diego, CA, USA), anti-mouse integrin α7-PE (130120812, Miltenyi Biotec, Bergisch Gladbach, Germany), and APC-Streptavidin (405207, BioLegend, San Diego, CA, USA). Cells were incubated at 4 °C for 30 min. Before the measurement, cells were washed with 0.5% BSA—physiological saline and suspended in 0.5% BSA—physiological saline supplemented with SYTO16 green, fluorescent nucleic acid stain (S7578, Invitrogen, Carlsbad, MA, USA) (5000× dilution) and SYTOX AADvanced dead cell stain (S10274, Invitrogen, Carlsbad, MA, USA) (1000× dilution) stains. For cell number determination, microparticles based on polystyrene, size 8 µm (78511, Sigma Aldrich, Budapest, Hungary), were used. Live cells were selected based on SYTO16 positivity and SYTOX AAD negativity; SCs were gated as CD45, CD31, Sca1, TER-119 negative, and integrin-α7^+^ cells. Fluorescent intensity was detected with an Agilent NovoCyte instrument (Agilent Scientific Instruments, Santa Clara, CA, USA). The magnetically separated muscle-derived CD45^+^ cells were stained with a combination of Alexa Fluor 488-conjugated anti-F4/80 antibody (MF48020, Invitrogen, Carlsbad, MA, USA) and Alexa Fluor 647-conjugated anti-Ly6G/Ly6C (GR-1) antibodies (108418, BioLegend, San Diego, CA, USA) at room temperature for 15 min. Cells were gated based on their forward and side scatter properties. Mφs were gated as GR-1^−^ and F4/80^+^, while neutrophils as F4/80^−^ and GR-1^+^ cells. F4/80^+^ Mφs were also analyzed for Ly6C, CD206, or MHCII expressions following staining with Ly6C PerCP-Cy5.5 (128012, BioLegend, San Diego, CA, USA), CD206-PE (141705, BioLegend, San Diego, CA, USA), or MHCII-FITC (107605, BioLegend, San Diego, CA, USA) antibodies, respectively. Fluorescent intensity was determined with a Becton Dickinson FACSCalibur instrument (Becton, Dickinson and Company, Franklin Lakes, NJ, USA).

### 2.11. C2C12 Cell Culture and Differentiation

Murine myoblast C2C12 cells were obtained from ATCC (CRL-1772), and cell culture was carried out as described earlier [26]. In brief, cells were grown in Dulbecco’s modified Eagle’s medium (DMEM) supplemented with 10% FBS, 100 U/mL penicillin, and 100 µg/mL streptomycin (growth medium) at 37 °C in 5% CO_2_ and 95% air at 100% humidity. For gene expression analysis, cells were plated into 24-well plates, while for immunofluorescent staining into 96-well plates at a density of 3500 cells/cm^2^. For the 6-day differentiation period, DMEM medium containing 2% FBS and 1% ITS (insulin, transferrin, sodium selenite) was used and replaced every 2nd day with a fresh one. In some experiments, the TG2 substrate biotin-cadaverine (100 µM) or the TG2 inhibitors ZDON (50 µM) or monodansyl cadaverine (100 µM) were added to the wells every 2nd day. Cells were labeled with MYHC4 antibody and DAPI to assess myoblast fusion, as described before [25]. ImageJ software was used to analyze digitally acquired photographs. The fusion index was obtained by expressing the number of nuclei within MYHC4-positive myotubes with ≥3 nuclei as a percentage of the total nuclei (*n* = 500), and additionally, the length of fibers was also measured. PrestoBlue (ThermoFisher Scientific, Waltham, MA, USA) staining was used to determine cell numbers according to the manufacturer’s recommendations. Fluorescence was detected on a SynergyTM H1 microplate reader (BioTek Instruments, Winooski, VT, USA). Propidium iodide (80 µg/mL, 5 min) was used to label dying cells in culture, while DAPI staining was used to determine total cell number.

### 2.12. In Vitro Phagocytosis Assay

Phagocytosis experiments were carried out as described previously [27]. Briefly, target C2C12 cells were induced to undergo necrosis by heating the cells at 65 °C for 10 min. C2C12 cells were labeled with 1µM CellTracker Deep Red Dye (ThermoFisher, Waltham, MA, USA) and added to µφs at 5:1 ratio (dead cell/Mφ). After 1 h co-culture, non-engulfed cells were washed away extensively, and µφs were detached by using EDTA. µφs were labeled with Alexa Fluor 488 conjugated anti-F4/80 antibody (Invitrogen, Carlsbad, MA, USA), and the percentage of engulfing cells was determined on a Becton Dickinson FACSCalibur flow cytometer (Becton, Dickinson and Company, Franklin Lakes, NJ, USA).

### 2.13. Western Blot Analysis

Western blot was carried out as described previously [25]. The whole-cell homogenate was used to detect MYHC4 in C2C12 cells and GDF3 in muscle-derived CD45^+^ cells. The homogenate was prepared in ice-cold lysis buffer (10% *v/v* glycerol, 1% *v/v* Triton X-100, 1 mM EGTA, 20 mM Tris,100 µM β-glycerophosphate, 137 mM NaCl, 5 mM EDTA, 1.04 mM AEBSF, 0.8 µM aprotinin, 40 µM bestatin, 14 µM E-64, 20 µM leupeptin and 15 µM pepstatin A, pH 7.9). The protein content of the samples was determined by Bio-Rad Protein Assay Dye (Bio-Rad, Budapest, Hungary), and then, the samples were boiled in a loading buffer with an aliquot corresponding to 40 µg of protein. After electrophoresis and blotting, protein bands were visualized by anti-GDF3 (cat#: AF958) (R&D Systems, Minneapolis, MN, USA) and anti-MYHC4 (cat#: 53-6503-82) (Thermo Fisher Scientific, Waltham, MA, USA) antibodies. Equal protein loading was demonstrated by probing the membranes with anti-lamin B (cat#: sc-6216) or anti-β-actin (cat#: A5441) antibodies (Santa Cruz Biotechnology, Dallas, TX, USA).

### 2.14. Quantification of Necrotic Areas

Necrotic myofibers were defined as pink, pale patchy fibers that are infiltrated by basophil single cells and quantified as described previously [28]. Briefly, 4 non-overlapping microscope view field areas were digitally acquired from 6–8 H&E-stained TA muscle sections at 200-fold magnification. The proportion of necrotic area/total regenerating area was determined after the manual outlining the necrotic fibers in the sections.

### 2.15. Statistical Analysis

All data presented represent the result of at least three independent experiments, and all data are presented as dots or mean or median ± SD or ± SEM. Statistical analysis was performed using a two-tailed, unpaired Student’s t-test and ANOVA with a post hoc Tukey HSD test. The equal variance of the sample groups was tested by F-test. * denotes *p <* 0.05, ** denotes *p <* 0.01.

## 3. Results

### 3.1. TG2 Deficiency Impairs Skeletal Muscle Differentiation and Function

**Figure 1:**
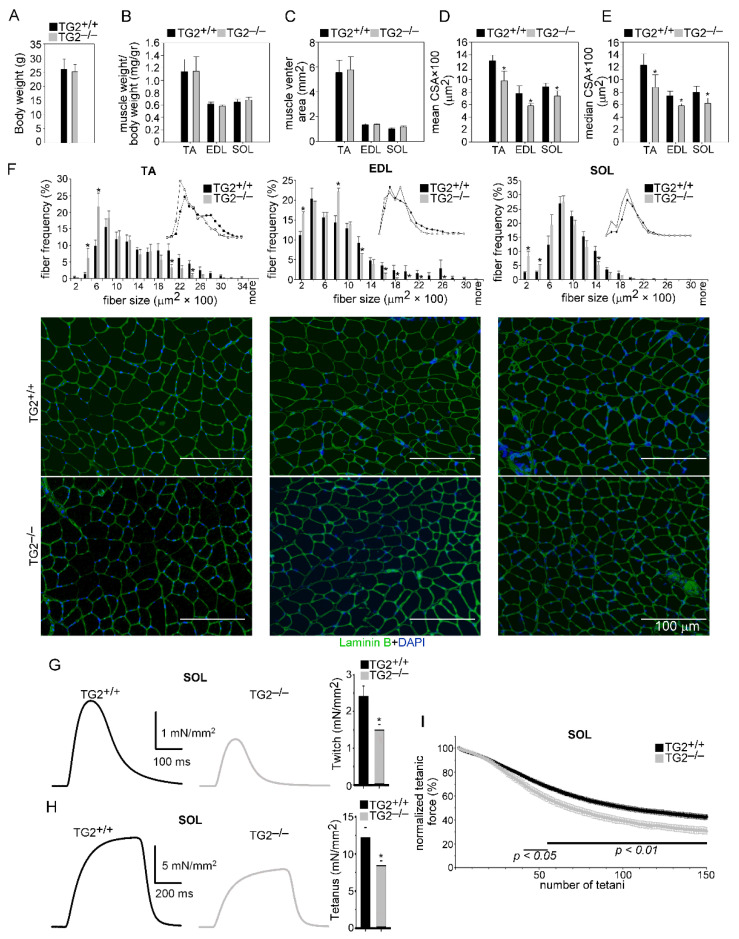
Loss of TG2 impairs muscle development and function. (**A**) Body weights of TG2^+/+^ and TG2^−/−^ mice. (**B**) The ratio of muscle weight/body weight, (**C**) muscle venter area, (**D**) mean myofiber cross-sectional area, and (**E**) median myofiber cross-sectional area (CSA) of TA, EDL, and SOL muscles in TG2^+/+^ and TG2^−/−^ mice. Data are expressed as mean or median ± SD. (**F**) Distribution of myofiber sizes in control TA, EDL, and SOL muscles of TG2^+/+^ and TG2^−/−^ mice with their representative immunofluorescence images of laminin (green) and DAPI (blue) nuclear staining. Data are expressed as mean ± SEM (*n* = 6). In total, 500 or more myofibers were analyzed in each sample using ImageJ software. Scale bars, 100 µm. (**G**) Representative and averaged peak ex vivo twitch and (**H**) tetanic force in soleus muscle from TG2^+/+^and TG2^−/−^ mice stimulated at 0.5 or 100 Hz, respectively, at room temperature (23–25 °C). The force was normalized to the cross-sectional area of the muscle. (**I**) Fatigue of SOL muscle was evoked with 150 tetani at a frequency of 0.5 Hz, and the amplitude of consecutive tetani was normalized to first tetanus developed. Solid horizontal lines below the data points represent the interval where data are significantly different from TG2^+/+^. The number of animals and muscles investigated is given in Table 1. Asterisks indicate statistically significant difference (* *p <* 0.05, Student’s *t*-test).

To study the role of TG2 in muscle homeostasis, we compared the characteristics of the tibialis anterior, extensor digitorum longus, and soleus muscles from TG2^+/+^ and TG2^−/−^ mice. There was no significant difference between the body weights, TA, EDL, and SOL muscle weights, and sizes of muscle venters between TG2^+/+^ and TG2^−/−^ mice (Figure 1A–C). However, a significantly smaller mean and median fiber CSA in muscles of TG2^−/−^ mice were detected as compared to wild-type ones (Figure 1D,E). The CSA frequency distribution showed that the frequency of smaller fibers was higher, while that of bigger fibers was lower in all the three TG2^−/−^ muscles as compared to wild-type ones (Figure 1F).

As loss of TG2 resulted in smaller myofiber CSAs in the skeletal muscle, we decided to determine whether this phenomenon has an impact on the physical performance of TG2^−/−^ mice. In vivo grip force is a measure of muscular strength and can be used as a tool to study the upper body and overall strength. Thus, we measured grip force in 18–20-week-old TG2^+/+^ and TG2^−/−^ mice. The maximal force of TG2^−/−^ animals was significantly smaller than that of TG2^+/+^ animals (139.37 ± 5.47 and 122.28 ± 4.52 mN in wild-type versus TG2 null muscles, respectively; significantly different at *p <* 0.05, *n* = 7). Since the average body weight was identical in both groups, the normalized grip force was also significantly smaller in TG2^−/−^ animals (4.92 ± 0.14 and 4.27 ± 0.15 mN/g in wild-type versus TG2 null muscles, respectively; significantly different at *p <* 0.01, *n* = 7).

To clarify the origin of this decrease in force, the ex vivo force was investigated in detail in the fast (glycolytic) EDL and the slow (oxidative) SOL muscles. There was no significant difference in the mean amplitude and the time course of the normalized single twitches and tetani of EDL muscles from TG2^−/−^ and TG2^+/+^ animals (Table 1). However, we observed a decreasing tendency in the maximal EDL muscle force of TG2^−/−^ mice (Table 1).

On the other hand, both the twitch (Figure 1G) and tetanic (Figure 1H) force decreased significantly in the soleus of TG2^−/−^ animals (Table 2). However, we did not find significant differences between the time to peak and half relaxation time of the same muscles (Table 1 and Table 2). The fatigability of both EDL and SOL muscles was also investigated by eliciting 150 consecutive tetanus. Similar to the single contractions, we observed significant differences in fatigue only in the SOL muscle. This muscle showed faster fatigue in the case of TG2^−/−^ mice than in TG2^+/+^ littermates, with the fatigue being significantly more pronounced from the 40th tetanus (Figure 1I, Table 2). This difference was not present in the case of EDL muscle (Table 1).

### 3.2. Normal Histological Appearance of Regenerating TA Muscles in the Absence of TG2

**Figure 2:**
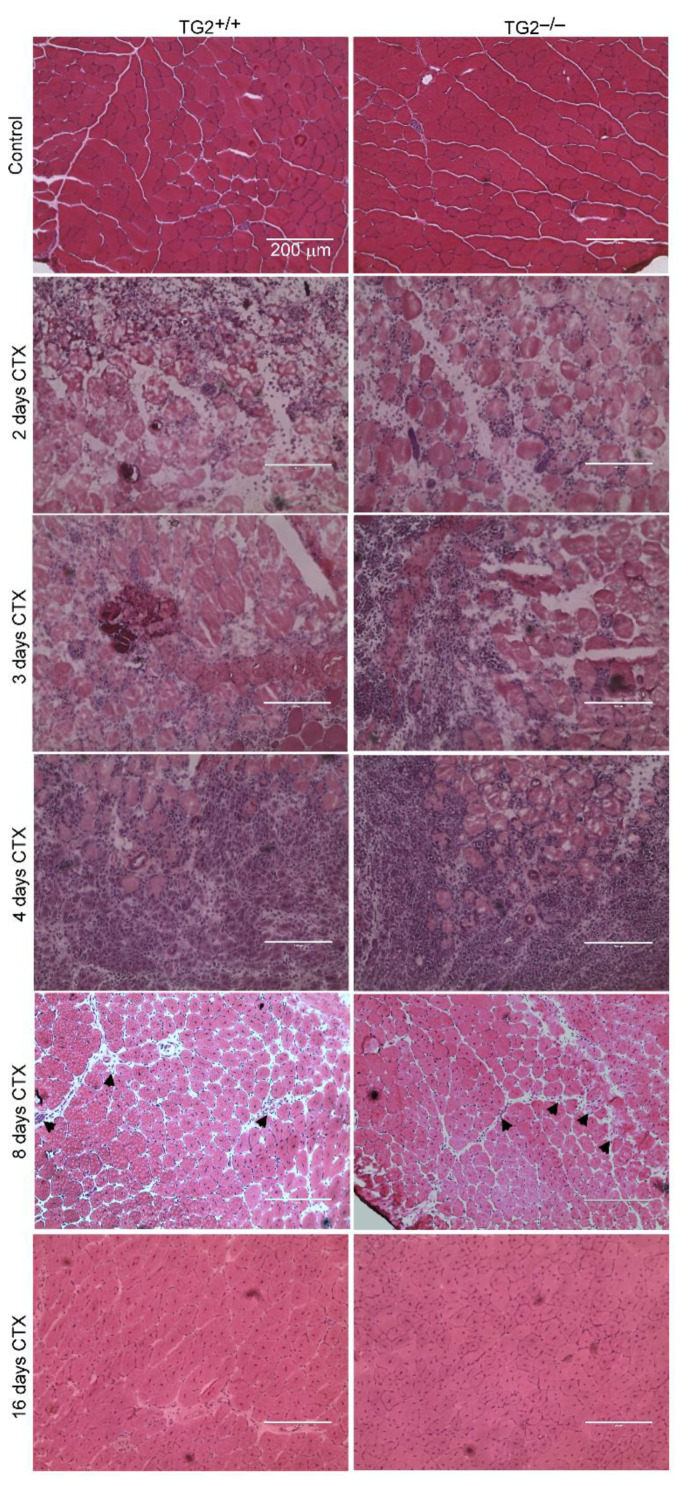
Time-dependent histological morphology of TA muscles following cardiotoxin-induced (CTX) injury in TG2^+/+^ and TG2^−/−^ mice. Muscle injury was induced by injecting 50 µL of 12 µM CTX into the TA muscles. Representative H&E-stained cross-sections of TG2^+/+^ and TG2^−/−^ TA muscles without treatment and at 2, 3, 4, 8, and 16 post-CTX–induced injury (*n* = 4 in each group). Arrows indicate necrotic areas at day 8. Scale bars, 200 µm.

**Figure 3:**
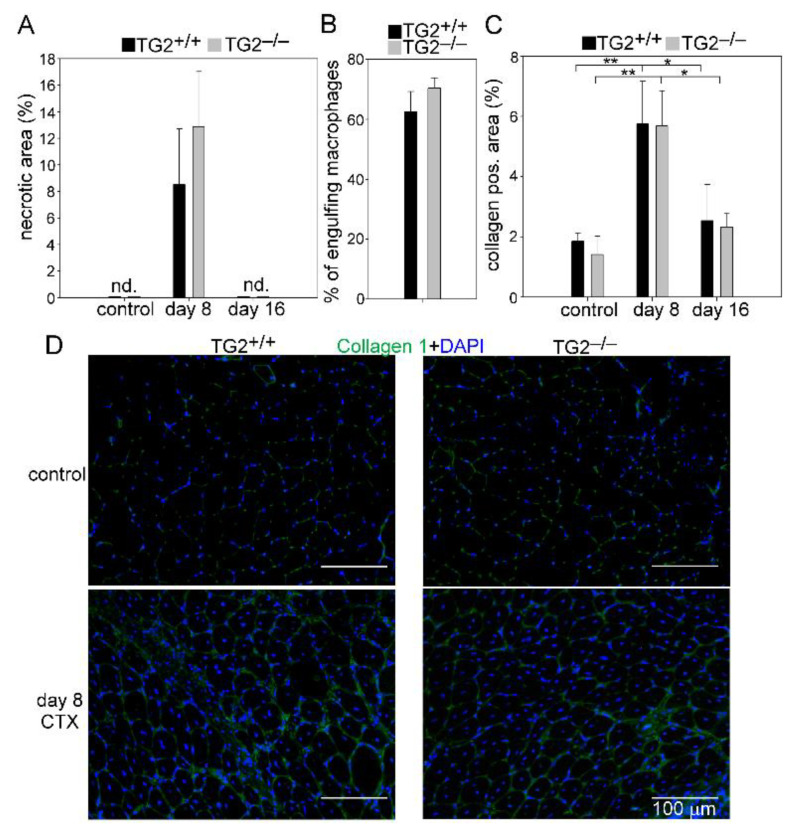
Unaffected necrotic cell clearance and collagen deposition in TA muscles following CTX-induced injury in TG2^−/−^ mice. (**A**) Quantification of necrotic areas in the control and in the regenerating muscles of TG2^+/+^ and TG2^−/−^ mice at day 8 and 16 post-CTX–induced injury. (**B**) Phagocytic capacity of F4/80^+^ cells isolated from regenerating muscles at day 4 post-injury. (**C**) Quantification of collagen 1 positive areas in the control and in the regenerating muscles of TG2^+/+^ and TG2^−/−^ mice at day 8 and 16 post-CTX–induced injury. (**D**) Representative immunofluorescence images of type 1 collagen (green) and DAPI (blue) nuclear staining in control and in TG2^+/+^ and TG2^−/−^ TA muscles regenerating for 8 days. All data are expressed as mean ± SD (*n* = 4). Asterisks indicate statistically significant difference (* *p <* 0.05, ** *p <* 0.01, ANOVA-test).

To study the role of TG2 in muscle regeneration, we performed histological analysis of control and CTX injected TA muscles of wild-type and TG2^−/−^ mice. There was no obvious difference in the gross appearance between the control muscles (Figure 2). On days 2, 3, and 4, both TG2^+/+^ and TG2^−/−^ regenerating muscles displayed local necrosis and abundant inflammatory cell infiltration. By day 8, most of the necrotic fibers were cleared from the muscles, and by day 16 post-injury, the overall histological architecture of both TG2^+/+^ and TG2^−/−^ muscles had been largely restored, and necrotic fibers were no longer visible (Figure 2 and Figure 3A). Since we and others have reported that TG2 is involved in the phagocytosis of apoptotic cells [13,16,29], we compared the phagocytic capacity of TG2^+/+^ and TG2^−/−^ muscle-derived macrophages. In line with the similar necrotic areas in TG2^+/+^ and TG2^−/−^ muscles, we did not observe any difference between the efferocytic capacity of TG2 deficient Mϕs (Figure 3B).

Efficient muscle repair requires the temporary deposition of extracellular matrix proteins. We detected an increased amount of collagen 1 in the regenerating muscles of both TG2^+/+^ and TG2^−/−^ mice as compared to their own non-regenerating muscles, but there was no significant difference between the two strains (Figure 3C,D).

### 3.3. TG2 Deficiency Impairs TA Muscle Regeneration

**Figure 4:**
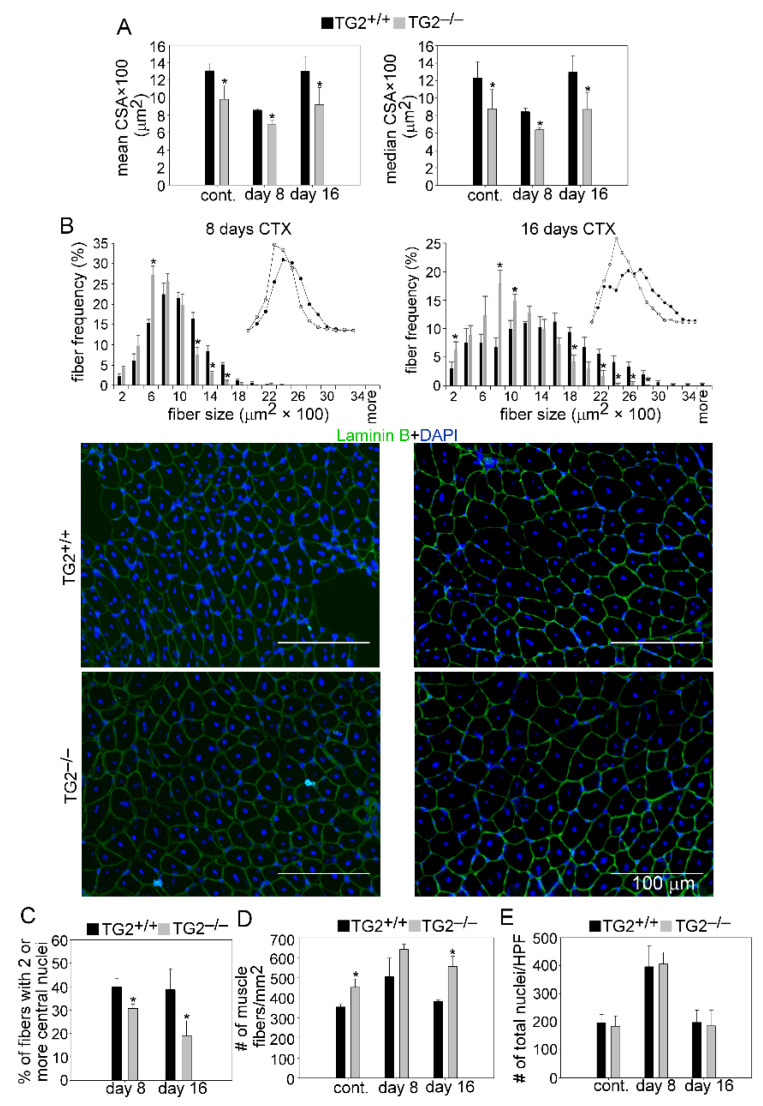
Muscle regeneration is impaired in TG2^−/−^ mice. (**A**) Mean and median myofiber cross-sectional areas (CSA), and (**B**) distribution of myofiber sizes in TA muscles of TG2^+/+^ and TG2^−/−^ male mice regenerating for 8 and 16 days following CTX-induced injury together with their representative immunofluorescence images of laminin (green) and DAPI (blue) nuclear staining. Scale bars, 100 µm. In total, 500 or more myofibers were analyzed in each sample using ImageJ version 1.52p software. (**C**) Percentage of newly formed myofibers containing two or more central nuclei and (**D**) number (#) of muscle fibers in control and regenerating TA muscles of TG2^+/+^ and TG2^−/−^ mice and at day 8 and 16 post-CTX-induced injury. (**E**) The number of nuclei counted on high power field (HPF) images of laminin and DAPI stained TA muscle sections from control and regenerating TA muscles of TG2^+/+^ and TG2^−/−^ mice. Data are expressed as mean ± SD except for fiber distribution analysis where mean ± SEM in case of (*n* = 6). Asterisks indicate statistically significant difference (* *p <* 0.05, Student’s *t*-test).

To study further a possible role of TG2 in muscle regeneration, we compared the myofiber cross-section areas of control and CTX-treated TA muscles from TG2^+/+^ and TG2^−/−^ mice. Similar to the non-injected control muscles, the mean and median CSA of newly formed myofibers with central nuclei in TG2^−/−^ mice were also significantly smaller than in TG2^+/+^ mice at day 8 and 16 post-injury (Figure 4A). The CSA frequency distribution showed that the frequency of smaller fibers was higher, while that of bigger fibers was lower in control and regenerating TG2^−/−^ muscles as compared to wild-type ones (Figure 4B).

The number of myofibers with two or more central nuclei is an indicator of myoblast fusion in the regenerating muscles. The number of newly formed fibers with two or more central nuclei was decreased significantly in TG2^−/−^ mice as compared to wild-type mice at days 8 and 16 post-injury (Figure 4C). Similar to the control muscles, we detected a significantly increased number of fibers in the TG2^−/−^ TA muscles at days 8 and 16 post-injury as well (Figure 4D), while the number of total nuclei was similar in the control and regenerating muscles of TG2^+/+^ and TG2^−/−^ mice (Figure 4E). Together, these data suggest a delayed regeneration and improper myoblast fusion in the absence of TG2.

### 3.4. Normal Proliferation and Differentiation of Satellite Cells (SCs) after Injury in the Absence of TG2

To assess a possible effect of TG2 ablation on SC cell proliferation and differentiation and on gene expression in the regenerating TA muscles, we followed the time-dependent changes in the number of SCs.

**Figure 5:**
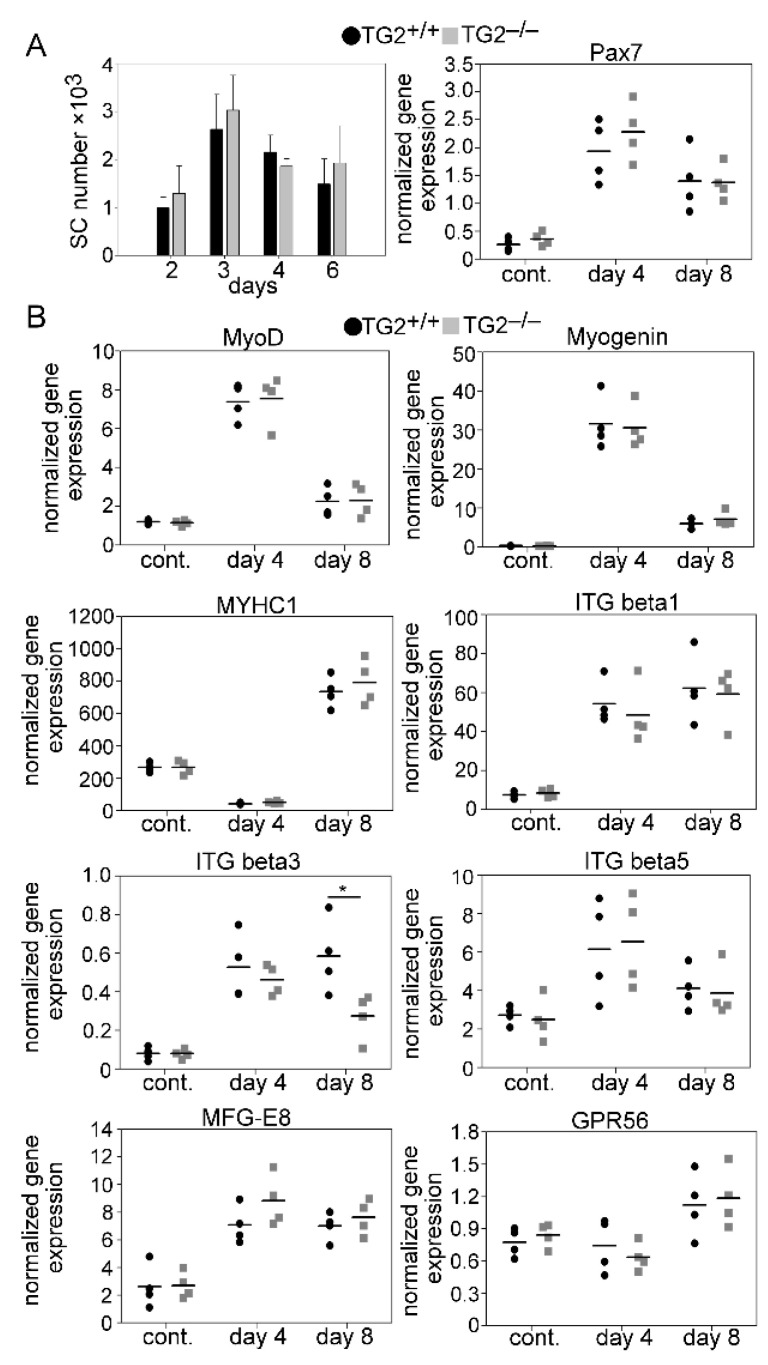
The number of SCs and expression of myogenic genes in the TA muscle of wild-type and TG2 null mice. (**A**) Number of SCs and mRNA expressions of Pax7 in non-injected control and regenerating TA muscles of TG2^+/+^ and TG2^−/−^ mice. (**B**) mRNA expressions of myogenic genes in control and CTX-injured TA muscles regenerating for 4 and 8 days determined by qRT-PCR analysis. Data are expressed as mean ± SD in Figure 1A left, while dots represent data from individual animals (*n* = 4). Asterisks indicate statistically significant difference (* *p <* 0.05, Student’s *t*-test).

In addition, the time-dependent changes in the skeletal muscle mRNA expressions of myogenic genes, such as the Pax7, MyoD, and myogenin transcription factors involved in myoblast proliferation and differentiation, and that of the MYHC1 differentiation marker were also determined during muscle repair following CTX-induced injury. In addition, we also determined the mRNA expressions of extracellular TG2 interacting partners known to be involved in myoblast proliferation, differentiation, or fusion, such as milk fat globule epidermal growth factor 8 (MFG-E8) [30,31], G protein-coupled receptor 56 (GPR56) [32], and ITGβ1 [33], 3, and 5 [34]. As demonstrated in Figure 5, with the exception of integrin β3 expression at day 8 post-injury, loss of TG2 did not affect any of the investigated parameters implying that loss of TG2 does not directly influence the proliferation or the differentiation of SCs in the skeletal muscle.

### 3.5. Unaltered Recruitment of Mϕs and Neutrophils after Injury in the Absence of TG2

**Figure 6:**
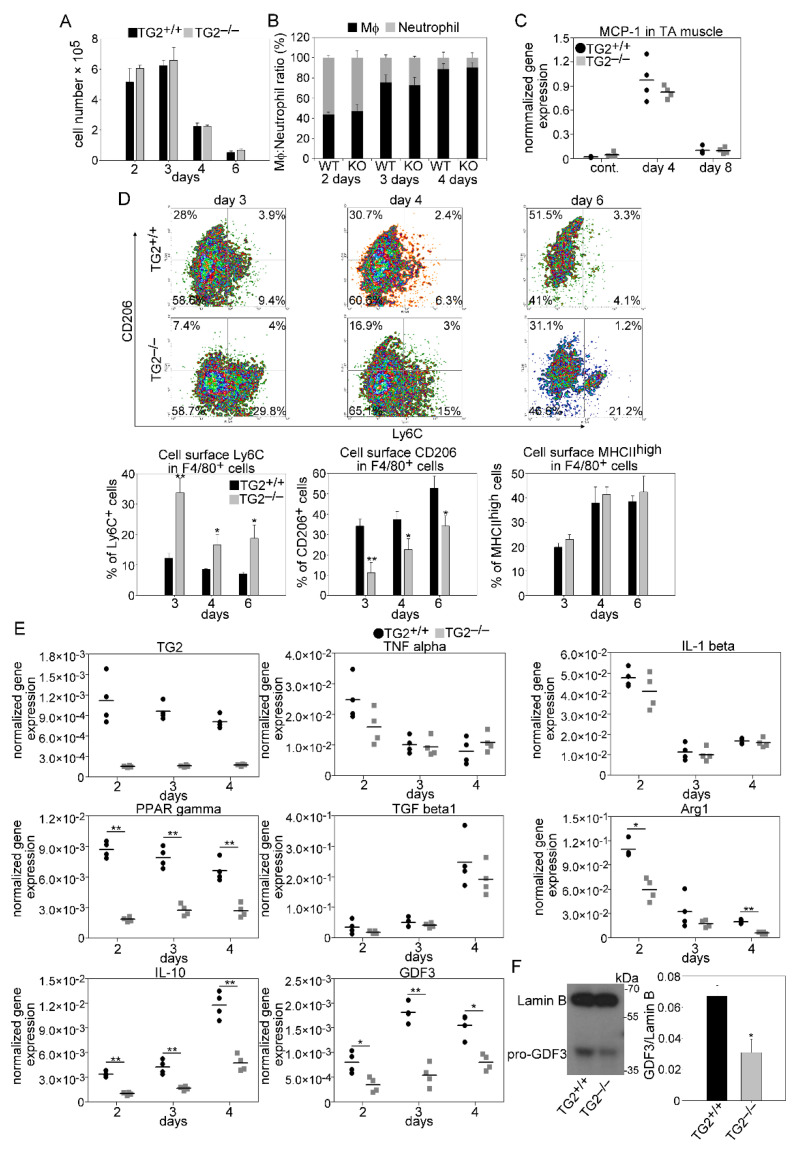
As compared to wild-type mice, leukocyte infiltration is not altered in TG2 null regenerating TA muscles following CTX injury, but the pro-inflammatory to healing phenotypic conversion of macrophages is delayed. (**A**) Number of CD45^+^ leukocytes per injured muscle and (**B**) ratio of anti-F4/80 antibody stained Mϕs and anti-Ly6G/Ly6C (GR-1) stained neutrophils within the CD45^+^ leukocyte population in TG2^+/+^ and TG2^−/−^ TA muscles during the first 4 days of regeneration following CTX-induced injury (*n* = 3). (**C**) MCP-1 mRNA expression levels of muscle-derived wild-type or TG2 null CD45^+^ leukocytes determined by qRT-PCR following CTX-induced injury (*n* = 4). (**D**) Representative scatter plots of CD206 and Ly6C stained muscle-derived F4/80^+^ cells and the percent of Ly6C^+^, CD206^+^, and MHCII^high^ cells within the muscle-derived F4/80^+^ population determined at the indicated days following CTX-induced injury in the TA muscles of TG2^+/+^ and TG2^−/−^ mice (*n* = 3). (**E**) mRNA expressions of TG2 and pro-and anti-inflammatory marker genes in CD45^+^ cells isolated from TA muscles determined by qRT-PCR at day 2, 3, and 4 post-injury (*n* = 4). (**F**) Protein level of GDF3 at day 4 post-injury in CD45^+^ cells isolated from regenerating TA muscles determined by Western blot analysis (*n* = 3). All data are expressed as mean ± SD, while dots represent data from individual animals. N in all these experiments corresponds to number of animals injured on both legs to harvest enough leukocytes from each mouse. Asterisks indicate statistically significant difference (* *p <* 0.05, ** *p <* 0.01, Student’s *t*-test).

Following injury, muscle repair is initiated by the migration of inflammatory cells to the injury site. To determine the composition of leukocytes in the early phase of muscle regeneration, we performed flow cytometric analysis of magnetically separated CD45^+^ cells from collagenase digested muscles. In accordance with previous observations, we detected early infiltration of neutrophils at day 2 post-injury followed by an increasing number of Mϕs at days 3 and 4 in wild-type mice. Loss of TG2 did not affect significantly the number of infiltrating CD45^+^cells (Figure 6A), the neutrophil/Mϕ ratios (Figure 6B), or the level of monocyte chemoattractant protein-1 (MCP-1) (Figure 6C), the main chemoattractant signal for Mϕ recruitment [35], in the regenerating muscles.

### 3.6. Impaired M1/M2 Phenotypic Switch in TG2 Null Macrophages during the Muscle Regeneration Process

The switch from a pro-inflammatory to an anti-inflammatory and healing environment around the myoblasts generated by the engulfing macrophages plays a key role during skeletal muscle regeneration. To investigate the impact of TG2 ablation on the phenotypic switch of macrophages and on the cytokine production, CD45^+^ cells from collagenase digested regenerating muscles were magnetically isolated at days 2, 3, and 4 post-injury, and cell surface stained for F4/80, Ly6C, CD206, and MHCII proteins. In addition, their gene expressions were also determined by quantitative PCR. To our surprise, though the loss of TG2 did not affect the efferocytotic capacity of muscle-derived macrophages (Figure 3A,B), still it delayed the generation of Ly6C^−^ CD206^+^ macrophages from the Ly6C^+^ pro-inflammatory cells. At the same time, it had no effect on the formation of the MHCII^high^ expressing cells (Figure 6D).

TG2 was expressed at the same level in wild-type CD45^+^ cells at all investigated time points during the regeneration (Figure 6E). We did not detect any difference in the expressions of the M1-like Mϕ-specific IL-1β and TNFα pro-inflammatory cytokines between TG2^+/+^ and TG2^−/−^ CD45^+^ cells, but peroxisome proliferator-activated receptor (PPAR)γ, a transcription factor that contributes to the M1/M2 like conversion of macrophages [7], was expressed at a significantly lower level in the TG2^−/−^ CD45^+^ cells than in the wild–type ones. From the investigated M2-like specific genes, TGFβ showed no difference, while the arginase 1 (Arg1) and IL-10 expressions were significantly lower in the TG2^−/−^ cells as compared to the wild-type cells.

Moreover, we also detected decreased M2-specific GDF3 mRNA expression and protein levels (Figure 6F) at all investigated time points in the TG2^−/−^ leukocytes, as compared to wild-type ones. Altogether these data indicate that TG2 promotes the formation of a subset or subsets of Ly6C^−^ Mϕs that are characterized by CD206, Arg1, IL-10, and GDF3 expressions, while has no effect on the downregulation of pro-inflammatory cytokines or on the formation of MHCII^high^ expressing Mϕs.

### 3.7. TG2 Promotes Myoblast Fusion without Using Its Crosslinking Activity

**Figure 7:**
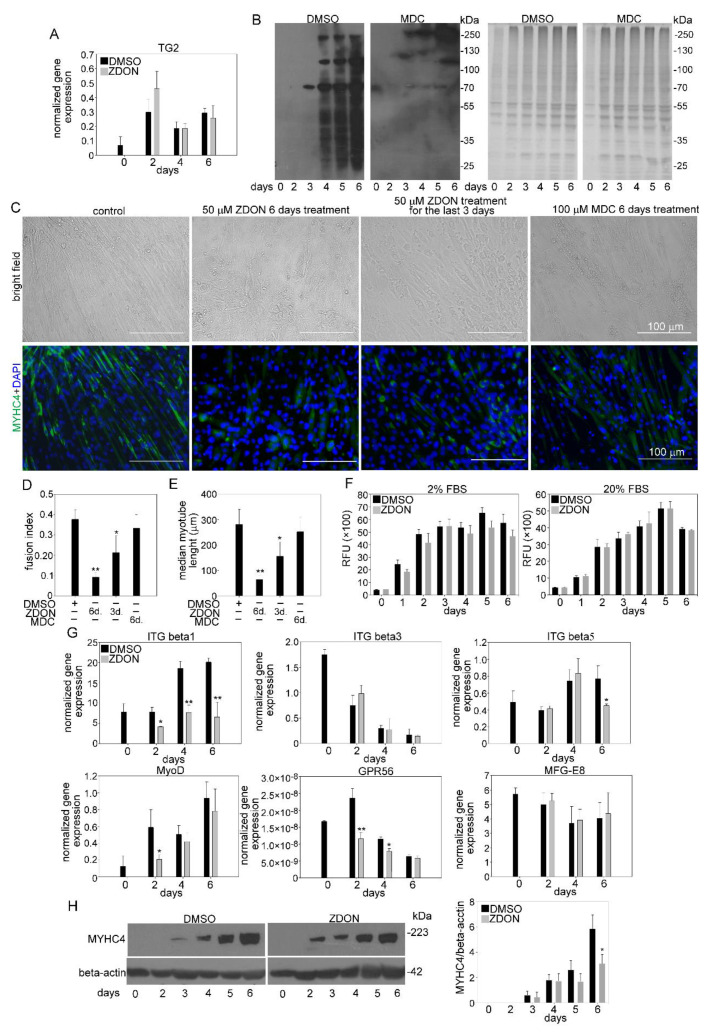
TG2 promotes fusion of C2C12 myoblasts without using its crosslinking activity. (**A**) mRNA expression levels of Table 2. in differentiating C2C12 myoblasts in the presence or absence of 50 µM ZDON, a conformation inhibitor of TG2 crosslinking activity, added at day 0 of differentiation (**B**) Biotin-cadaverine incorporation into proteins of differentiating C2C12 myoblasts in the presence and absence of 100 µM monodansylcadaverine, a competitive inhibitor of TG2 crosslinking activity, detected by Western blot analysis. Protein loadings on the Coomassie dye-stained gel are also shown. (**C**) Representative light- and fluorescent microscopic images of C2C12 myoblasts differentiated for 6 days in the absence or presence of 50 µM ZDON added at day 0 or day 4 of differentiation, or in the presence of 100 µM monodansylcadaverine added at day 0 of differentiation. MYHC4 was visualized by using anti-MYHC4 antibody (green) and nuclei by DAPI (blue). Scale bars, 100 µm. (**D**) Fusion index of C2C12 cells differentiated for 6 days in the absence and presence of TG2 inhibitors. (**E**) The length of myotubes generated from C2C12 myoblasts differentiated for 6 days in the absence or presence of TG2 inhibitors (**F**) Alterations in the number of viable C2C12 cells grown in growth and differentiation medium in the presence or absence of 50 µM ZDON added at day 0 determined by PrestoBlue staining. (**G**) mRNA expression levels of myogenic genes in differentiating C2C12 myoblasts in the presence or absence of 50 µM ZDON (added at 0 day of differentiation) determined by qRT-PCR. (**H**) Protein expression levels of myosin heavy chain 4 (MYHC4) in differentiating C2C12 myoblasts in the presence or absence of 50 µM ZDON (added at day 0 of differentiation) determined by Western blot analysis. β-actin was used as a loading control. One representative blot of three is shown. All the data are expressed as mean ± SD of three independent experiments. Asterisks indicate statistical significance (* *p <* 0.05, ** *p <* 0.01, Student’s *t*-test).

Transferring mouse C2C12 myoblasts from growth medium to low-serum fusion medium triggers the generation of multinucleated, myosin-expressing myotubes [36] and provides a quantifiable in vitro model of myogenesis [37]. We applied this model to study whether TG2 is required for the myogenesis process. As shown in Figure 7A, initiation of myoblast differentiation induced the mRNA expression of TG2 by day2, then it gradually declined. Increased expression of TG2 was accompanied by increased TG2 crosslinking activity detected by the enhanced incorporation of a biotinylated primary amine (5-{[(N-biotinoylamino)hexanoyl]amino}pentylamine(biotin-x-cadaverine) into proteins from day 4 of differentiation (Figure 7B).

To investigate the effect of the loss of TG2 on myoblast differentiation, we exposed C2C12 myoblasts to ZDON, a cell-permeable, irreversible active-site inhibitor of TG2 [38], which is able to trap TG2 in the open conformation (the structure is deposited at PDB (3S3J). This inhibitor was selected because it is efficiently taken up by cells, and it inhibits both the crosslinking activity of the enzyme and its interactions with other proteins that are not formed if TG2 is in its open conformation. As seen in Figure 7C, after a 6-day differentiation period, C2C12 cells formed large multinucleated myotubes, which show positive staining for the muscle-specific marker MYHC4. Exposure to ZDON decreased the capacity of C2C12 cells to form multinucleated myotubes and decreased the length of myotubes (Figure 7D,E). This inhibition was not due to direct cytostatic or cytotoxic effects of ZDON since it did not decrease the C2C12 cell proliferation (Figure 7F) in the differentiation (2% FBS) or growth medium (20% FBS), and it did not increase the percentage of propidium iodide positive dying cells either (data not shown). ZDON treatment did not affect the mRNA expressions of TG2, ITGβ3, or MFG-E8, but it decreased the mRNA expressions of MyoD, ITGβ1, and Gpr56 (Figure 7G). By day 6, we also detected a lower protein expression of MYHC4 in ZDON-exposed differentiating C2C12 cells by Western blot analysis (Figure 7H). ZDON was also effective if it was added only from day 4 of differentiation when the crosslinking activity of TG2 became detectable, but its effect was less pronounced (Figure 7C).

To determine whether the crosslinking activity of TG2 is required for the observed effect of TG2 inhibition on myotube formation, differentiating C2C12 cells were exposed to monodansylcadaverine (MDC). MDC is a competitive inhibitor of the enzyme on the endogenous substrates, though it is also an alternative substrate. Previous studies, however, have shown that it interferes with biological processes that depend on the crosslinking activity of TG2 without altering the conformation of the enzyme. Thus, MDC allowed us to study the consequences of the loss of the TG2 crosslinking activity selectively [39]. As seen in Figure 7C, MDC applied in a concentration at which it efficiently inhibited the crosslinking activity of TG2 (Figure 7B) had no effect on myoblast fusion (Figure 7C–E). Altogether our data indicate that TG2 is required for proper myoblast fusion without a need for its crosslinking activity.

## 4. Discussion

Skeletal muscle regeneration following injury results from the proliferation and differentiation of myogenic stem cells located beneath the basal lamina of the muscle fibers. Satellite cell differentiation is not a satellite cell-autonomous process but depends on signals provided by the surrounding cells. Infiltrating macrophages play a determining role in the process partly by removing the necrotic cell debris, partly by releasing cytokines and growth factors that drive myogenesis. The early pro-inflammatory cytokines promote SC proliferation and differentiation, while the growth factors are needed to promote myoblast fusion and growth. Proper efferocytosis plays a determining role in the process because it not only leads to the removal of cell debris but also facilitates the phenotypic switch of macrophages, thus the switch in the production of the myogenesis-regulating cytokines and growth factors as well.

Previous studies have already indicated that impaired efferocytosis related to the loss of various phagocytic receptors or their transcriptional regulators might lead to impaired muscle regeneration. Thus, the loss of scavenger receptor BI [28], Mer [25], or PPARγ [7] all resulted in a delay in the phenotypic switch of engulfing muscle macrophages and consequently in a delayed muscle repair. Though TG2 has been shown to participate in efferocytosis by certain macrophage types [13,16,29], the loss of it, similar to RPE cells [40], did not affect the efferocytic capacity of infiltrating skeletal muscle macrophages determined either in vivo or in vitro. However, its lack still resulted in an impaired macrophage phenotypic switch. Though the loss of both Mer and TG2 delayed the conversion of the Ly6C^+^ pro-inflammatory Mϕs to Ly6C^−^ M2 like Mϕs, loss of Mer affected the formation of MHC^high^ cells [25], while loss of TG2 had no effect on these events. Instead, loss of TG2 delayed the rise in the CD206 mannose receptor [41], Arg1, GDF3, and IL-10-producing cells. Interestingly, the expression of all these factors is known to be regulated by PPARγ [7,42,43], a transcription factor playing a central role in the phenotypic switch of macrophages. Accordingly, we detected significantly lower PPARγ mRNA levels in the CD45^+^ cells derived from theTG2 null regenerating TA muscles. Our data indicate that TG2 might affect the phenotypic switch of macrophages by influencing the expression levels of PPARγ.

Skeletal muscle fibers are syncytia that are generated via the fusion of myoblasts to form multinucleated myotubes. Myoblast fusion is initiated by an alignment of myoblast and/or myotube membranes, followed by rearrangements of the actin cytoskeleton at the contact sites, and then membrane fusion. In mammals, it occurs during embryogenesis and in the adult, promoting generation, growth, and repair of muscle fibers [44]. While impaired phagocytosis of apoptotic cells might cause a delay in myoblast fusion during muscle repair, it does not affect the development of the skeletal muscle. Thus, both Mer null [25] and scavenger receptor BI null [28] mice have a normal skeletal muscle architecture despite the fact that they show impaired muscle regeneration following injury. On the other hand, skeletal muscles of mice that have impaired myoblast fusion, such as myoferlin [45] or stabilin-2 [46] null ones, are characterized by small size muscle fibers. Since TG2 null skeletal muscles are also built up from small size myofibers, we turned to C2C12 myoblasts to see if TG2 also affects myoblast fusion.

The expression of TG2 did rise following the initiation of myoblast differentiation, while we detected an increase in its crosslinking activity at a later stage of differentiation concomitant with the start of the myoblast fusion process. Still, inhibition of its crosslinking activity had no effect on myoblast fusion. In accordance, previous studies have also demonstrated that the crosslinking activity of TG2 is not required for myoblast fusion but it contributes to the myofibril assembly within the generated myotubes [21,22]. However, when such a TG2 inhibitor was applied, which in addition to inhibiting its crosslinking activity also trapped the conformation of the protein in its open form, an inhibition of myoblast fusion and growth was observed.

Increasing evidence indicates that TG2 participates in protein/protein interactions in its closed guanine-bound conformation. For example, TG2 mutants, which could not be secreted or could not bind guanine nucleotides, were not able to replace the wild-type protein in its integrin coreceptor function [13,29]. Integrin β1, β3, and β5 were all shown to participate in myoblast fusion [33,34]. These receptors, however, do not mediate the membrane fusion itself. Rather, they participate in the prefusion events, such as myoblast differentiation [47,48], cell-cell recognition, adhesion, and cytoskeletal rearrangements, which are needed to generate and then bring the fusion proteins in proper orientation and proximity between the two fusing cells. Integrin β3 and Rac activity, for example, are required for both myoblast differentiation and adhesion [34], but they need to be downregulated prior to fusion [49]. Accordingly, similar to β3 integrin-silenced myoblasts [34], we detected decreased MyoD expressions in ZDON-treated C2C12 cells. TG2 not only acts as a coreceptor for integrin function but also interacts with their ligand MFG-E8 [13]. MFG-E8 was shown to be released from myoblasts [30], and we found that its mRNA expressions significantly increased during muscle regeneration together with that of the integrins. Altogether these data indicate that TG2 might promote myoblast fusion directly acting as a coreceptor for myoblast integrins. In addition, based on a previous report, it might also contribute to myogenesis acting as a ligand for Gpr56 during later phases of the process [50], though the loss of Gpr56 alone did not cause muscle developmental defects [32], while the loss of integrin β1 did [33].

We also investigated whether alteration in the skeletal muscle structure in TG2 animals affects their physical performance. We could prove in living animals and in isolated soleus muscles that the deletion of TG2 causes decreased force production. This effect was accompanied by faster fatigue in the soleus. At the same time, the lack of TG2 did not affect the functional properties of an almost completely fast muscle as the EDL [51]. Since both muscles are characterized by small size myofibers, the observed difference in the physical performance seems to be related to their different metabolic phenotype rather than to the altered skeletal muscle structure. While slow muscles are built up from highly oxidative fibers that are capable of performing prolonged low-intensity activities, fast muscles contain highly energy-consuming fibers that depend mainly on anaerobic metabolism [52]. Previous studies have demonstrated that TG2 acting in the mitochondria promotes the function of the electron transport chain and consequently ATP production in the cardiac muscle [53] and also in the forebrain [54]. If this role of TG2 is general, then the loss of it might explain our observation on the soleus muscle, the physical activity of which is strongly dependent on the mitochondria.

Altogether, our results reported in this paper indicate that TG2 expressed by both macrophages and myoblasts contributes to proper myoblast fusion, and its ablation leads to impaired muscle development and regeneration in mice.

## Figures and Tables

**Table 1:** Ex vivo measurement of EDL muscle force and fatigue. Fatigue was followed during 150 consecutive tetanus.

	TWITCH		TETANUS	
	TG2^+/+^	TG2^−/−^	TG2^+/+^	TG2^−/−^
Number of animals	8	7	8	7
Number of muscles	14	11	14	11
Muscle weight (mg)	17.41 ± 0.91	16.61 ± 0.55	-	-
Force (mN/mm^2^)	1.75 ± 0.24	1.47 ± 0.12	8.37 ± 1.31	7.61 ± 0.63
TTP (ms)	32.7 ± 1.0	31.0 ± 0.6	186.9 ± 7.4	186.7 ± 5.0
HRT (ms)	26.5 ± 1.4	24.5 ± 1.4	74.4 ± 8.2	75.7 ± 5.1
Duration (ms)	205.6 ± 33.3	181.2 ± 25.8	344.6 ± 8.8	343.0 ± 5.2
Muscle venter area (mm^2^)	1.36 ± 0.08	1.37 ± 0.06	-	-
Fatigue at 50 (%)	-	-	33.8 ± 1.8	37.2 ± 2.3
Fatigue at 100 (%)	-	-	63.7 ± 1.8	63.6 ± 2.1
Fatigue at 150 (%)	-	-	72.9 ± 1.8	76.4 ± 1.8

TTP—time to peak; HRT—half relaxation time.

**Table 2:** Ex vivo measurement of soleus muscle force and fatigue. Fatigue was followed during 150 consecutive tetanus.

	TWITCH		TETANUS	
	TG2^+/+^	TG2^−/−^	TG2^+/+^	TG2^−/−^
Number of animals	8	7	8	7
Number of muscles	13	11	13	11
Muscle weight (mg)	18.32 ± 1.24	19.48 ± 1.31	-	-
Force (mN/mm^2^)	2.41 ± 0.28	1.49 ± 0.16 *	12.18 ± 1.36	8.42 ± 0.66 *
TTP (ms)	77.9 ± 3.8	77.1 ± 1.6	520.4 ± 1.4	522.9 ± 1.1
HRT (ms)	73.5 ± 5.6	72.4 ± 5.1	106.9 ± 3.8	105.7 ± 3.4
Duration (ms)	314.2 ± 24.4	301.4 ± 17.9	766.4 ± 10.0	771.5 ± 18.1
Muscle venter area (mm^2^)	1.02 ± 0.09	1.18 ± 0.08	-	-
Fatigue at 50 (%)	-	-	29.3 ± 0.5	38.4 ± 3.0 *
Fatigue at 100 (%)	-	-	49.6 ± 0.6	60.7 ± 2.5 **
Fatigue at 150 (%)	-	-	57.5 ± 0.6	69.1 ± 2.9 **

* and ** show significant differences TG2+/+ at *p <* 0.05 and *p <* 0.01, respectively. TTP—time to peak; HRT—half relaxation time.

## Data Availability

The data presented in this study are available upon request from the corresponding author.
